# Supplementary material for: Penetration Efficiency of Antitumor Agents in Ovarian Cancer Spheroids: The Case of Recombinant Targeted Toxin DARPin-LoPE and the Chemotherapy Drug, Doxorubicin
Source: Pharmaceutics. 2019 May 7;11(5):219. doi: 10.3390/pharmaceutics11050219 (PMC6572593; doi:10.3390/pharmaceutics11050219)
Supplement: Supplementary file 1 [file pharmaceutics-11-00219-s001.pdf]

pharmaceutics

MDPI

Supplementary Materials: Penetration Efficiency of Antitumor Agents in Ovarian Cancer Spheroids: The Case of Recombinant Targeted Toxin DARPin-LoPE and the Chemotherapy Drug, Doxorubicin

Evgeniya Sokolova, Olga Kutova, Alena Grishina, Anton Pospelov, Evgeniy Guryev, Alexey Schulga, Sergey Deyev and Irina Balalaeva

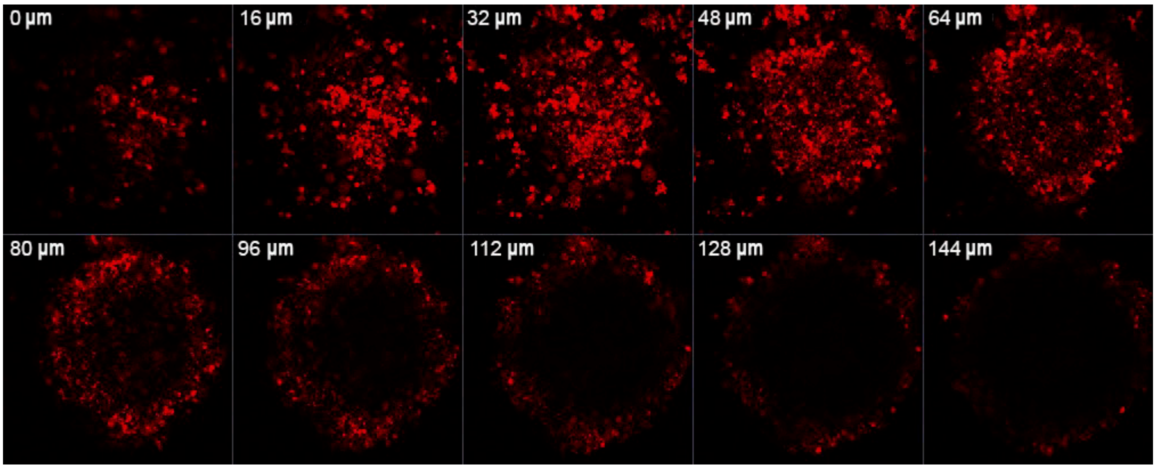

Figure S1. Sequential optical sections of SKOV-3 spheroid after incubation for 24 hours with DARPin-LoPE, labeled with a red fluorescent dye DyLight650. Step - 16 µm. Image size is 708 × 708 µm.

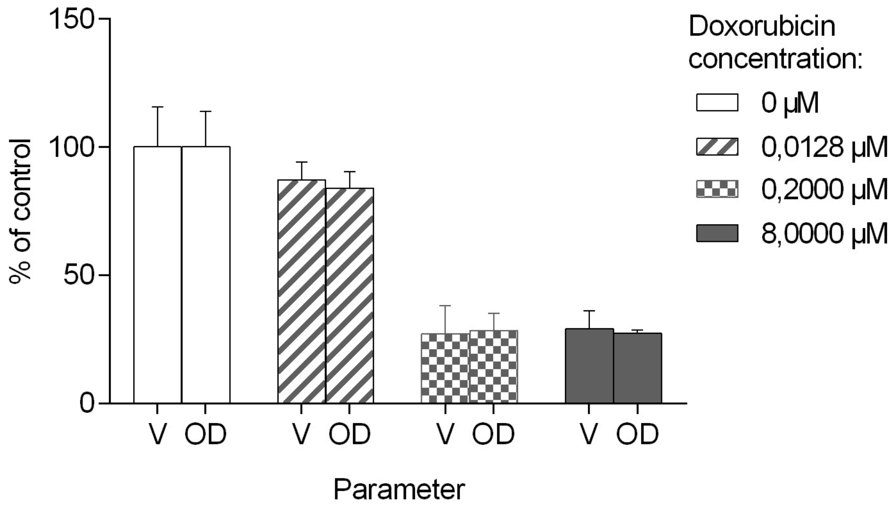

Figure S2. Assessment of the doxorubicin cytotoxicity using two approaches: by the standard MTT test ("OD") and using the size (volume) of the spheroids at the end of incubation with the agent ("V"). Spheroids were incubated with doxorubicin for 7 days. Pearson's correlation coefficient r = 0.97, $p < 0.0001$.

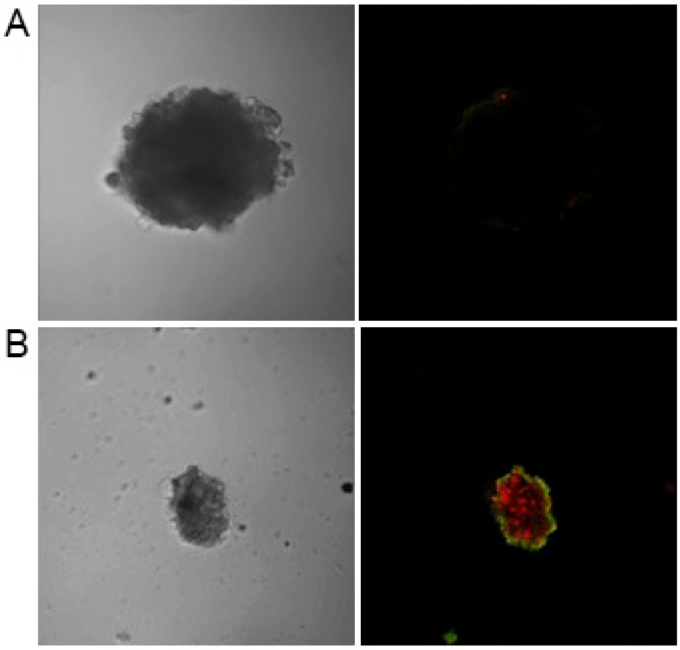

Figure S3. Preliminary study of cell death in SKOV-3 spheroids under DARPin-LoPE treatment. The SKOV-3 spheroids were incubated in absence (control, **A**) or in presence of 1 μM DARPin-LoPE (**B**) for five days, stained with BD FITC AnnexinV Apoptosis detection kit (cat. No. 556547) and imaged by confocal microscopy immediately.

Images in transmitted light (on the left) and merged images in green and red channels (on the right) are presented. Green channel (Annexin-FITC): λex 488 nm, λem 517–595 nm. Red channel (Propidium iodide): λex 488 nm, λem 600–670 nm. Image size is 708 × 708 μm.

A cell dying via apoptosis is typically stained with Annexin V-FITC (at the initial stages) or with both Annexin V-FITC and Propidium iodide (at the later stages of apoptotic death). We observed staining with Propidium iodide throughout the entire volume of the treated spheroids, while Annexin V-FITC was localized only on the spheroids surface. Apparently, it is impossible to interpret the results of such experiments correctly when working with living intact spheroids, because the question about the penetration of dyes into the spheroid arises. Propidium iodide being a low-molecular-weight compound (~0.65 kDa), seems to penetrate well into the spheroid, while penetration of the Annexin protein (~36 kDa) is most likely difficult.
